# Draft Genome Sequences of 15 Bacterial Species Constituting the Stable Defined Intestinal Microbiota of the GM15 Gnotobiotic Mouse Model

**DOI:** 10.1128/MRA.00686-20

**Published:** 2020-08-27

**Authors:** Céline Elie, Alban Mathieu, Adrien Saliou, Adrien Villain, Marion Darnaud, François Leulier, Andrea Tamellini

**Affiliations:** a BIOASTER, Institut de Recherche Technologique, Lyon, France; b Institut de Génomique Fonctionnelle de Lyon, Université de Lyon, Ecole Normale Supérieure de Lyon, Centre National de la Recherche Scientifique, Université Claude Bernard Lyon 1, Lyon, France; University of Rochester School of Medicine and Dentistry

## Abstract

The GM15 community is a bacterial consortium used to generate a novel standardized mouse model with a simplified controlled intestinal microbiota recapitulating the specific opportunistic pathogen-free (SOPF) mouse phenotype and the potential to ensure an increased reproducibility and robustness of preclinical studies by limiting the confounding effect of microbiota composition fluctuation.

## ANNOUNCEMENT

The intestinal microbiota is a complex and dynamic ecosystem largely composed of bacteria whose activity greatly impacts the health and diseases of the host (1). Associating mice with stable defined bacterial consortia reduces the complexity of the microbiota and overcomes limitations related to the variability between individuals and animal facilities (2, 3). Therefore, gnotobiotics contribute to standardization and experimental reproducibility and are a powerful tool for testing causality in host-microbiome studies (4–6). Thus, we have developed a simplified mouse microbiota that is representative of the fecal microbiota found in C57BL/6J mice on the functional level and derived a standardized gnotobiotic mouse model called GM15, which has been bred successfully for over eight generations in the gnotobiology unit of BIOASTER. All animal procedures were approved by the French Ministry of Higher Education, Research and Innovation (MESR) and the ANSES/ENVA/UPEC ethics committee (Autorisation de Projet Utilisant des Animaux à des Fins Scientifiques [APAFIS] no. 4529-2016022616404045v3, 785-2015042819315178v2, and 18918-2019020118003843v3) and were conducted in accordance with national French and European legislation on the protection of animals used for scientific purposes.

We report here the draft genome sequences of 9 bacterial strains isolated from the intestinal microbiota of C57BL/6J specific-opportunistic-pathogen-free (SOPF) mice (Charles River Laboratories, France), 2 bacterial strains isolated from C57BL/6J axenic mice recolonized with feces of the altered Schaedler flora (ASF) mouse model (Taconic, USA), and 4 bacterial strains obtained from the DSMZ collection. Then, the colonization of the axenic C57BL/6J mice with these 15 bacterial isolates resulted in the GM15 mice.

Fresh cecal contents and fecal pellets of mice were resuspended (1/10 [wt/vol]) in reduced broth medium for direct dilution plating onto agar plates (same medium as the broth) and growth at 37°C under an anaerobic atmosphere (90% N_2_, 5% H_2_, 5% CO_2_). Lactobacillus johnsonii MD006 was isolated on MRS agar. Lactobacillus murinus MD040 and Parabacteroides goldsteinii MD072 were isolated on Columbia nalidixic acid (CNA) agar with 5% sheep blood. Bacteroides acidifaciens MD185 and *Lachnospiraceae* sp. strain MD308 were isolated on Gifu anaerobic medium (GAM) agar. Bacteroides caecimuris MD237 and Lactobacillus reuteri MD207 were isolated on GAM agar supplemented, respectively, with 32 μg/ml vancomycin and 32 μg/ml erythromycin. *Lachnospiraceae* sp. strains MD335 and MD329 were isolated on M2GSC (modified Med2 of Hobson) agar (7). All isolates mentioned were isolated from fecal pellets of SOPF mice, except for isolates MD335, MD329, and MD308, which were isolated from the cecal contents of SOPF mice. *Clostridium* sp. strain MD294 and *Schaedlerella arabinosiphila* strain MD300 were isolated on M2GSC agar (7) from the cecal contents and fecal pellets of ASF-colonized mice, respectively. Prior to genome sequencing, bacteria were identified to the genus or species level by 16S sequencing.

Genomic DNA of each bacterial isolate was extracted using the DNeasy PowerLyzer PowerSoil kit (Qiagen) from a culture inoculated with a single bacterial colony at 37°C in an anaerobic chamber (90% N_2_, 5% H_2_, 5% CO_2_), except Escherichia coli DSM-28618, which was grown aerobically. The bacterial strains were grown in modified GAM broth (HyServe), except for isolates MD300, MD335, MD329, and MD308, which grew better in M2GSC medium (7), and Anaerotruncus colihominis DSM-28734, which was grown in *Bifidobacterium* medium (DSMZ, medium 58). DNA samples from the 15 bacterial cultures were prepared for whole-genome sequencing using the Nextera XT DNA library preparation kit (Illumina). The resulting libraries were analyzed using the high-sensitivity DNA kit on the Agilent 2100 Bioanalyzer system and quantified using the QuantiFluor One double-stranded DNA (dsDNA) kit (Promega). Paired-end (2 × 300-bp) sequencing was performed on a MiSeq sequencer using the MiSeq v3 kit (600 cycles; Illumina). The paired-end reads were assembled *de novo* using the A5-miseq assembly pipeline (8), comprising the following steps: adapter trimming, quality trimming and filtering, error correction, assembly, and scaffolding. The 15 *de novo* assemblies resulted in draft genome sequences composed of few scaffolds with high *N*_50_ values. Genomes were then ordered using Mauve (9) and annotated with the NCBI Prokaryotic Genome Annotation Pipeline (PGAP) (10). Default parameters were used for all software tools.

Following Edgar’s recommendation (11), a full-length 16S rRNA sequence identity ≥99% using either the BLAST (12), RDP (13), or EzTaxon (14) programs allowed the identification of 12 isolates at the species level. Despite phylogenetic placements and average nucleotide identity (ANI) calculations performed with GTDB-Tk (15), isolates MD329, MD335, and MD308 could only be assigned to the *Lachnospiraceae* family.

These 15 bacteria cover 7 of the most representative and prevalent families of the intestinal microbiota of C57BL/6J SOPF mice (3).

### Data availability.

The assembled sequences and sequencing reads have been deposited in DDBJ/ENA/GenBank and SRA, respectively (BioProject accession no. PRJNA551571), under the accession numbers listed in Table 1. The versions described in this paper are the first versions.

**TABLE 1:** Accession numbers and characteristics of genomes of the GM15 bacterial consortium

Isolate	Bacterial species	GenBank accession no.	SRA accession no.	Assembly size (bp)	No. of paired-end reads	GC content (%)	Genome coverage (×)	No. of contigs	*N*_50_^*a*^ (bp)	Genome completeness^*b*^					
										C (%)	S (%)	D (%)	F (%)	M (%)	*n*
MD185	*Bacteroides acidifaciens*	VIRE00000000	SRR9696648	5,228,881	3,643,154	42.03	209	174	78,435	100.0	99.8	0.2	0.0	0.0	541
MD237	*Bacteroides caecimuris*	VIRD00000000	SRR9696649	4,824,756	3,072,634	41.91	191	93	111,017	100.0	99.8	0.2	0.0	0.0	541
MD294 (ASF356)	*Clostridium* sp.	VIRC00000000	SRR9696650	2,856,932	3,858,506	31.67	405	30	199,176	99.6	98.5	1.1	0.0	0.4	264
MD300 (ASF502)	*Schaedlerella arabinosiphila*	VIRB00000000	SRR9696651	6,346,609	2,802,822	43.21	132	186	90,162	100.0	100.0	0.0	0.0	0.0	264
MD335	Unclassified *Lachnospiraceae* sp.	VIRA00000000	SRR9696652	5,174,557	3,623,726	42.12	210	189	74,501	100.0	99.6	0.4	0.0	0.0	264
MD006	*Lactobacillus johnsonii*	VIQZ00000000	SRR9696653	1,938,179	4,121,376	35.70	638	43	79,219	99.5	99.0	0.5	0.2	0.3	402
MD040	*Lactobacillus murinus*	VIQY00000000	SRR9696654	2,224,369	3,265,244	39.77	440	130	35,268	99.5	99.3	0.2	0.2	0.3	402
MD207	*Lactobacillus reuteri*	VIQX00000000	SRR9696655	2,014,265	3,994,740	39.72	595	55	13,099	99.5	99.0	0.5	0.2	0.3	402
MD072	*Parabacteroides goldsteinii*	VIQW00000000	SRR9696656	6,910,815	3,871,748	42.38	168	115	109,394	100.0	99.6	0.4	0.0	0.0	541
MD329	Unclassified *Lachnospiraceae* sp.	VIQV00000000	SRR9696657	2,789,948	3,468,474	39.51	373	86	103,195	98.9	98.9	0.0	0.0	1.1	264
MD308	Unclassified *Lachnospiraceae* sp.	VIQU00000000	SRR9696644	4,443,317	2,742,248	40.71	185	162	89,086	98.9	98.9	0.0	0.0	1.1	264
DSM-28734 (JM4-15)	*Anaerotruncus colihominis*	VIQT00000000	SRR9696645	3,502,576	2,247,686	53.63	193	37	200,988	97.3	97.3	0.0	0.4	2.3	264
DSM-26114 (YL32)	*Clostridium clostridioforme*	VIQS00000000	SRR9696646	7,084,105	4,288,290	45.80	182	268	69,589	99.2	97.3	1.9	0.4	0.4	264
DSM-1551 (I50)	*Clostridium cocleatum*	VIQR00000000	SRR9696647	3,057,667	3,798,362	29.16	373	53	110,672	95.5	95.0	0.5	0.5	4.0	218
DSM-28618 (Mt1B1)	*Escherichia coli*	VIQQ00000000	SRR9696643	5,218,283	1,968,758	40.69	113	33	943,892	100.0	99.5	0.5	0.0	0.0	440

a The *N*_50_ value is the length of the shortest contig for which longer and equal-length contigs cover at least 50*%* of the assembly.

b Genome completeness was assessed with BUSCO v4.0.6 (16) and is reported in BUSCO notation: C, complete; S, complete and single copy; D, complete and duplicated; F, fragmented; M, missing; *n*, total number of BUSCO groups searched.
